# Transcriptome analysis reveals effects of leukemogenic SHP2 mutations in biosynthesis of amino acids signaling

**DOI:** 10.3389/fonc.2023.1090542

**Published:** 2023-01-30

**Authors:** Yuming Zhao, Zhiguang Chang, Bingbing Hu, Qi Zhang, Dengyang Zhang, Chunxiao He, Yao Guo, Zhiyong Peng, Chun Chen, Yun Chen

**Affiliations:** ^1^ Edmond H. Fischer Translational Medical Research Laboratory, Scientific Research Center, The Seventh Affiliated Hospital, Sun Yat-sen University, Shenzhen, Guangdong, China; ^2^ Reproductive Medicine Center, The Seventh Affiliated Hospital, Sun Yat-sen University, Shenzhen, Guangdong, China; ^3^ Nanfang-Chunfu Children’s Institute of Hematology, Taixin Hospital, Dongguan, Guangdong, China; ^4^ Department of Pediatrics, The Seventh Affiliated Hospital, Sun Yat-Sen University, Shenzhen, Guangdong, China

**Keywords:** leukemia, SHP2 mutations, metabolism, transcriptome, biosynthesis of amino acids

## Abstract

Gain-of-function mutations of SHP2, especially D61Y and E76K, lead to the development of neoplasms in hematopoietic cells. Previously, we found that SHP2-D61Y and -E76K confer HCD-57 cells cytokine-independent survival and proliferation *via* activation of MAPK pathway. Metabolic reprogramming is likely to be involved in leukemogenesis led by mutant SHP2. However, detailed pathways or key genes of altered metabolisms are unknown in leukemia cells expressing mutant SHP2. In this study, we performed transcriptome analysis to identify dysregulated metabolic pathways and key genes using HCD-57 transformed by mutant SHP2. A total of 2443 and 2273 significant differentially expressed genes (DEGs) were identified in HCD-57 expressing SHP2-D61Y and -E76K compared with parental cells as the control, respectively. Gene ontology (GO) and Reactome enrichment analysis showed that a large proportion of DEGs were involved in the metabolism process. Kyoto Encyclopedia of Gene and Genome (KEGG) pathway enrichment analysis showed that DEGs were the mostly enriched in glutathione metabolism and biosynthesis of amino acids in metabolic pathways. Gene Set Enrichment Analysis (GSEA) revealed that the expression of mutant SHP2 led to a significant activation of biosynthesis of amino acids pathway in HCD-57 expressing mutant SHP2 compared with the control. Particularly, we found that *ASNS*, *PHGDH*, *PSAT1*, and *SHMT2* involved in the biosynthesis of asparagine, serine, and glycine were remarkably up-regulated. Together, these transcriptome profiling data provided new insights into the metabolic mechanisms underlying mutant SHP2-driven leukemogenesis.

## Introduction

Src Homology 2 domain-containing protein tyrosine Phosphatase-2 (SHP2), encoded by *PTPN11* gene, is a classical non-receptor protein tyrosine phosphatase (PTP) (1). It is the first PTP recognized as an oncogene. SHP2 plays key roles in regulating RAS-ERK, PI3K-AKT, JAK-STAT and other signaling pathways, which are mainly downstream signals of growth factor, cytokine, and integrin receptors (2, 3). In general, SHP2 mutations are rare in solid tumors (3). Germline mutations in SHP2 present in ~50% of Noonan Syndrome and ~90% of LEOPARD syndrome, both congenital developmental disorders and mainly characterized by growth retardation, short stature, facial features, and heart defects (4). Somatic SHP2 mutations mainly occur in several types of hematologic malignancies, including ~10% myelodysplastic syndromes, ~5% juvenile acute myeloid leukemia, ~7% B-cell acute lymphoblastic leukemia, and particularly ~35% juvenile myelomonocytic leukemia (JMML) (3, 5–7). However, the molecular mechanisms of leukemogenesis driven by mutant SHP2 are not fully understood. Previous studies about SHP2 mutants mainly focus on the activation of tumor proliferation signaling pathways and the tumor microenvironment (1, 3). However, the effects of SHP2 mutants on cancer-cell metabolism have not been investigated. Characterizing the alterations in cellular biosynthesis can provide insights into the mechanism of mutant SHP2-driven leukemogenesis.

Various biological hallmarks of tumor cells are closely related to cell metabolism, including rapid proliferation, immune escape and drug resistance (8). The use of cellular nutrient generally requires the binding of growth factors to their receptors to activate a series of signaling pathways that initiate cell metabolism (9). However, gain-of-function mutations occur in growth factor receptors or downstream pathway genes in most tumor cells (10), leading to constantly activated signals that overcome the growth factor dependency (11). As the result, cancer cells acquire the ability to autonomously uptake nutrients, providing a material basis for the uncontrolled division and proliferation (9). Meanwhile, abnormal metabolic pathways often induce cancer cell-specific vulnerabilities, which provided potential therapeutic targets.

Metabolic reprogramming is believed to result from oncogene activation or metabolic enzymes alterations (12). Previous studies have shown that some key proteins in cell proliferation-related signaling pathways are involved in metabolic reprogramming (13). The serine/threonine kinase AKT, for instance, does not only activate cell division signals, but also regulates glucose uptake to provide energy to cancer cells. The activation of AKT has been found to support the growth factor-independent survival *via* multi-step regulation of glucose metabolism, including promotion of glucose uptake by up-regulation of glucose transporter 1 (GLUT1) and activation of hexokinase (HK) (14, 15). In most cases, a variety of oncogenes lead to metabolic reprogramming by inducing broad changes in gene expressions (16). For instance, MYC enhances aerobic glycolysis by up-regulating GLUT1, PKM, LDH and MCT1, which also reprograms the glutathione biosynthesis (13, 17). Besides, cancer cells with specific oncogenic activation exhibit a defined metabolic preference (16). For example, EGFR activation promotes the serine synthesis pathway whereas FGFR activation enhances aerobic glycolysis and recycles lactate (16).

Our previous studies have shown that the expression of mutant SHP2 led to growth factor-independency of HCD-57, an erythropoietin (EPO)-dependent erythroid leukemia cell line (18), suggesting the possibility of mutant SHP2-reprogrammed cell metabolism in HCD-57. For this reason, we investigated altered metabolism pathways of parental and SHP2-mutant HCD-57 based on transcriptome analysis. Analysis of Kyoto Encyclopedia of Gene and Genome (KEGG) metabolism-related pathways revealed that differentially expressed genes (DEGs) were mainly enriched in glutathione metabolism and biosynthesis of amino acids pathways. Gene Set Enrichment Analysis (GSEA) showed the biosynthesis of amino acids pathway was significantly activated by the expression of mutant SHP2. In addition, we found that mRNA expression of *ASNS* involved in asparagine synthesis, *PHGDH* and *PSAT1* involved in serine biosynthesis, and *SHMT2* involved in glycine synthesis were significantly increased in HCD-57 expressing mutant SHP2, compared with parental cells. Taken together, we identified aberrant metabolic pathways in mutant SHP2-driven leukemia cells, which may provide potential metabolism-targeted therapies for leukemia with SHP2 mutations.

## Materials and methods

### Cell culture

HCD-57 was a kind gift from Dr. Zhizhuang Joe Zhao, the University of Oklahoma, Health Science Center. HCD-57 was cultured in IMDM (Gibco, MA, USA) supplemented with 20% FBS (Hyclone, UT, USA) and 20 ng/mL EPO (Peprotech, NJ, USA). Parental HCD-57 cells were starved for 8 h without EPO before total RNA isolation. HCD-57/SHP2-D61Y and HCD-57/SHP2-E76K, as the mutant SHP2-expressing cells, have acquired EPO-independent survival and proliferation. They were cultured in IMDM supplemented with 20% FBS and in absence of EPO. All cells were cultured in a humidified atmosphere at 37°C with 5% CO_2_.

### Generation of mutant SHP2-transformed HCD-57

Retroviruses carrying mutant SHP2 were generated by using pMSCV-IRES-GFP as described previously (19). Briefly, the full-length SHP2-D61Y and SHP2-E76K were cloned to pMSCV-IRES-GFP, respectively. Plasmids containing mutant SHP2 were used to transfect GP2-293 cells together with pVSV-G helper plasmid using Lipofectamine 3000 reagent (Thermo Fisher Scientific, MA, USA). Subsequently, the medium was collected and centrifuged at 20, 000 g for 2 h at 4°C to enrich retroviruses. HCD-57 cells were infected by retroviruses with 5 μg/mL polybrene (Sigma-Aldrich, MO, USA) with centrifugation at 1, 800 g for 2 h at room temperature. The infected cells were cultured in IMDM in absence of EPO. Single colonies were picked after 8-10 days of culture and further expanded in EPO-free IMDM supplemented with 20% FBS.

### Total RNA isolation

Total RNA from cells was isolated using Trizol (Invitrogen, CA, USA) per the manufacturer’s instructions. The isolated total RNA was qualified and quantified by using a Nano Drop and Agilent 2100 bioanalyzer (Thermo Fisher Scientific, MA, USA).

### mRNA library construction

RNA-seq library construction and RNA high-throughput sequencing were entrusted to Beijing Genomics Institute (Beijing, China). In brief, mRNA for each sample was purified using Oligo(dT)-attached magnetic beads and then fragmented into small pieces with fragment buffer. First-strand cDNA was generated using random hexamer-primed reverse transcription, followed by a second-strand cDNA synthesis and end repair using A-Tailing Mix and RNA Index Adapters. The cDNA fragments were then amplified by PCR, and purified by Ampure XP Beads. The product was validated on the Agilent Technologies 2100 bioanalyzer. The double-stranded PCR products from previous step were heated denatured and circularized by the splint oligo sequence to get the final library, which was amplified with phi29 to make DNA nanoballs (DNBs) containing more than 300 copies of one molecular. DNBs were loaded into the patterned nanoarray and single end 50 bases reads were generated on BGIseq500 platform.

### Bioinformatics analysis

Clean reads were filtered by FASTQ (version 0.18.0). Reads containing sequencing adapters, unknown nucleotides (‘N’ base) and low-quality bases were removed. Clean reads were obtained and stored in FASTQ format. The clean reads were mapped to the reference genome using HISAT2 (v2.2.4). StringTie (V1.3.1) was applied to assemble the map reads and gotten fragments per kilobase of transcript per million mapped reads (FPKM) to calculated gene expression. Bioinformatic analyses were performed using the Omicsmart online platform (http://www.omicsmart.com). Principal component analysis (PCA) was performed with R package gmodels (http://www.r-project.org/). DEGs analysis was performed by edgeR. The parameter of false discovery rate (FDR) below 0.05 and absolute fold change ≥ 2 were considered DEGs. Analysis of Gene Ontology (GO), KEGG, and Reactome were based on database (http://www.geneontology.org/), (https://www.genome.jp/kegg/), and (https://reactome.org/). Enrichment analysis identified significantly enriched in DEGs comparing with the whole genome background. The calculated *P* value was gone through FDR correction, taking FDR ≤ 0.05 as a threshold. GSEA was performed using software GSEA to identify whether a set of genes in specific KEGG pathways shows significant differences in two groups.

## Results

### Transcriptomic profiling analyses of HCD-57 expressing SHP2-D61Y and -E76K

**Figure 1:**
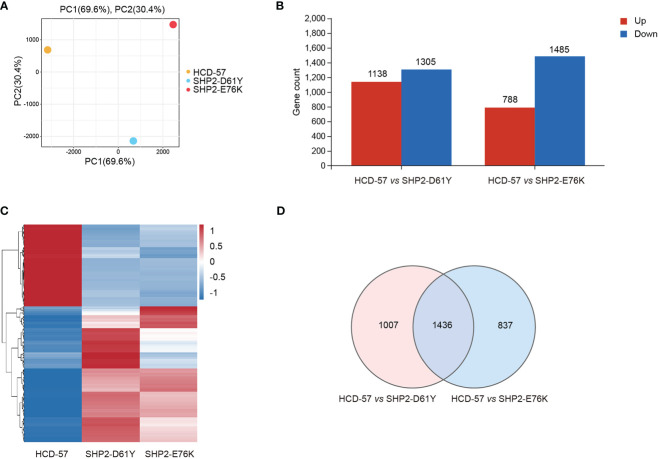
Basic transcriptomic analysis profile among mutant-SHP2 transfected cells and parental HCD-57 cells deprived EPO. **(A)** PCA plots of DEGs identified in HCD-57 cells expressing SHP2-D61Y and -E76K compared to parental HCD-57 cells. **(B)** The number of DEGs (up-regulated and down-regulated) in two compared groups. FDR<0.05, |log2FC|>1. **(C)** Heatmap of hierarchical clustering results for all identified DEGs at SHP2-D61Y, SHP2-E76K and parental HCD-57 cells (red, up-regulated; blue, down-regulated). **(D)** Venn diagram of the numbers of DEGs in HCD-57 vs SHP2-D61Y and HCD-57 vs SHP2-E76K.

To investigate the effect of mutant SHP2 on HCD-57 cells, RNA-seq was performed among HCD-57 cells expression SHP2-D61Y, SHP2-E76K, and parental HCD-57 cells. Owing to the expression of mutant SHP2 led to growth factor-independency of HCD-57, we cultured parental HCD-57 cells in medium deprived EPO for 8 h to remove the stimulation of growth factor. PCA analysis clearly separated the parental HCD-57 cells from HCD-57 expressing SHP2-D61Y and -E76K based on PC1, with PC1 contributing 69.6% variation, making it the dominant component (**Figure 1A**). The number of DEGs was 2443 and 2273 for HCD-57 cells expressing mutant SHP2-D61Y and -E76K compared to the control (**Figure 1B**), respectively. Hierarchical clustering of differential gene expression patterns was performed, and a heatmap was used to present the results. The analysis revealed comparable patterns among the HCD-57 cells expressing SHP2-D61Y and SHP2-E76K, while the transcriptome profiles of these mutant cells were much different from parental HCD-57 cells deprived of EPO (**Figure 1C**). A Venn diagram was performed and 1436 mutual DEGs were identified among the two compared groups (**Figure 1D**).

### Mutant SHP2 dysregulated cellular metabolic biological processes

**Figure 2:**
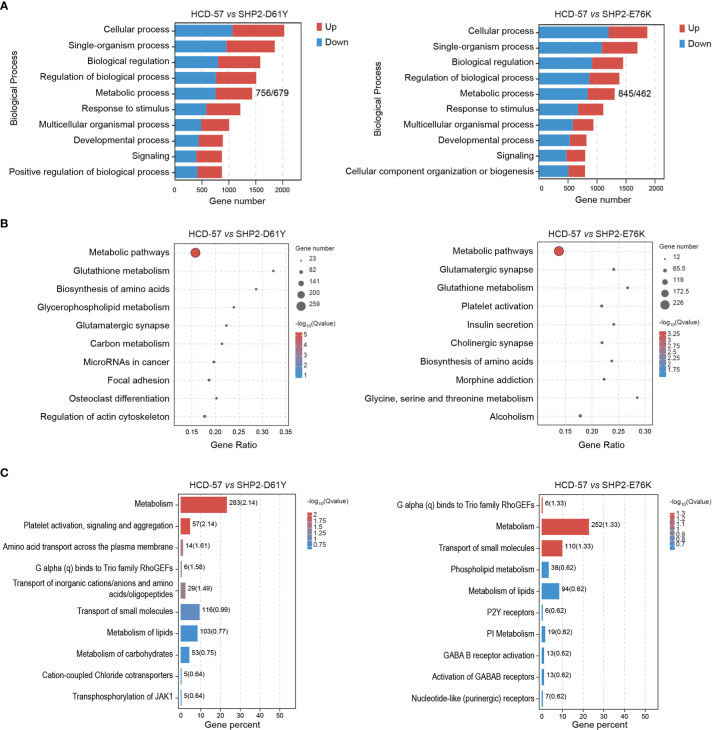
Enrichment analyses of all DEGs in HCD-57 vs SHP2-D61Y and HCD-57 vs SHP2-E76K. **(A)** Top 10 biological processes based on descending order by number of DEGs through GO analysis. **(B)** Top 10 pathways enriched by KEGG enrichment analysis. **(C)** Top 10 biological pathways and processes enriched by Reactome analysis.

We performed multiple enrichment analyses to investigate biological functions and altered pathways related to these DEGs. We found that a mass of dysregulation genes was related to metabolic process using GO classification in SHP2-D61Y and SHP2-E76K transformed cells compared to the control (**Figure 2A**). In cells expressing SHP2-D61Y, genes involved in metabolism accounted for ~60% in the total up-regulated DEGs, and ~58% in down-regulated DEGs, respectively. Cells transformed by SHP2-E76K showed ~59% for up-regulated proportion and ~57% for down-regulated proportion involved in metabolism. In addition, KEGG analysis in whole pathway maps identified metabolic pathways as the most significantly enriched pathways in both two comparison groups (**Figure 2B**). As expected, Reactome analysis also showed significant enrichment of metabolism reaction (**Figure 2C**). These results indicated that the expression of SHP2 mutants leads to a remodeling of cellular metabolism.

### Altered metabolism pathways in HCD-57 expressing mutant SHP2

**Figure 3:**
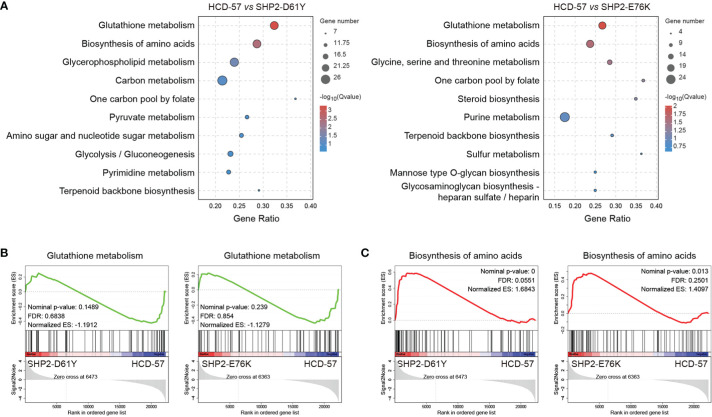
Pathways for metabolism-specific dysregulation caused by mutant SHP2 expression. **(A)** Top 10 enriched pathways related to metabolism based on KEGG enrichment analysis for all DEGs. GSEA plots of **(B)** Glutathione metabolism and **(C)** Biosynthesis of amino acids target genes on HCD-57 expressing SHP2-D61Y or -E76K vs parental cells. Normalized enrichment score (NES), nominal *P*-value and FDR Q-values are indicated.

We performed KEGG pathway analysis of all DEGs to identify significantly enriched metabolic pathways. We found that the most enriched pathways in cells expressing SHP2-D61Y and SHP2-E76K compared with the control were glutathione metabolism and biosynthesis of amino acids pathways (**Figure 3A**). Subsequently, we found that the expression of mutant SHP2-D61Y and SHP2-E76K significantly activated the biosynthesis of amino acids pathway based on GSEA analysis (**Figure 3C**). The schematic diagrams of alterations in KEGG pathways regarding biosynthesis of amino acids was revealed in **Supplementary Figures 1** and **2**. There was a down-regulation in the glutathione metabolism pathway whereas the nominal p-value and FDR q-value (false discovery rate) did not reach a statistical significance (**Figure 3B**). The remaining metabolic pathways that were significantly enriched (*P*<0.05) both in SHP2-D61Y and -E76K cells were presented in **Table 1**. These data suggest that biosynthesis of amino acids may play an important role in leukemogenesis induced by mutant SHP2.

**Table 1:** List of metabolic pathways significantly enriched both in HCD-57 cells expression SHP2-D61Y and -E76K (*P*<0.05).

Pathways		HCD-57 *vs* SHP2-D61Y				HCD-57 *vs* SHP2-E76K			
Name	KEGG-B-Class	DEGs	*P*-value	Q-value	NES (GSEA)	DEGs	*P*-value	Q-value	NES
Metabolic pathways	Global and overview maps	259	0.0000	0.0000	NA	226	0.0000	0.0004	NA
Glutathione metabolism	Metabolism of other amino acids	23	0.0000	0.0005	-1.1912	19	0.0001	0.0090	-1.1279
Biosynthesis of amino acids	Global and overview maps	23	0.0000	0.0030	1.6843	19	0.0004	0.0217	1.4097
Carbon metabolism	Global and overview maps	26	0.0014	0.0748	1.5362	19	0.0450	0.2511	1.2666
One carbon pool by folate	Metabolism of cofactors and vitamins	7	0.0041	0.1219	0.8887	7	0.0021	0.0486	-0.7613
Pyrimidine metabolism	Nucleotide metabolism	13	0.0124	0.2027	-0.8285	7	0.0093	0.1126	-0.8684
Glycine, serine and threonine metabolism	Amino acid metabolism	10	0.0200	0.2634	1.2259	12	0.0009	0.0302	1.0145
Sulfur metabolism	Energy metabolism	4	0.0309	0.3111	NA	4	0.0213	0.1769	NA
Purine metabolism	Nucleotide metabolism	23	0.0451	0.3758	0.8631	24	0.0074	0.1061	-0.9653

The threshold of significant differentially expressed genes (DEGs) was set as FDR<0.05, |log2FC|>1. *P*-value and Q-value were calculated from KEGG analysis. Normalized Enrichment Score (NES) were obtained from Gene Set Enrichment Analysis (GSEA).

### Genes associated with serine and glycine synthesis were significantly up-regulated in SHP2-mutant HCD-57

**Figure 4:**
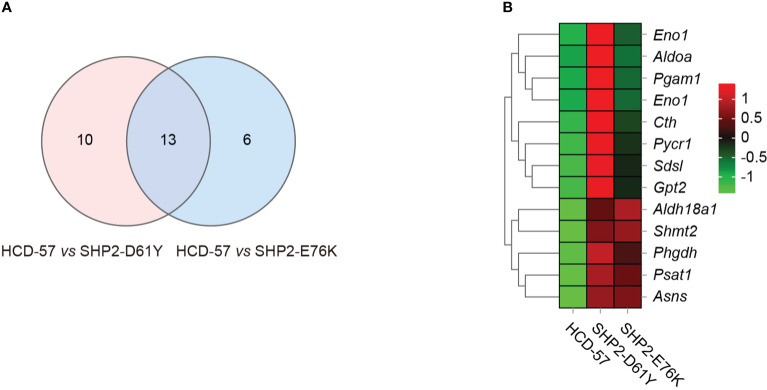
Differential gene analysis involved in the biosynthesis of amino acids pathway caused by mutant SHP2 expression. **(A)** Venn diagram indicating the overlap dysregulated genes in the biosynthesis of amino acids pathway in HCD-57 vs SHP2-D61Y and HCD-57 vs SHP2-E76K. **(B)** Heatmap of 13 DEGs shared in both comparisons in the biosynthesis of amino acids metabolites pathway.

To further investigate genes with significantly altered expression in the biosynthesis of amino acids caused by the expression of SHP2-D61Y and SHP2-E76K, we obtained 13 mutually dysregulated genes using the Venn diagram (**Figure 4A**). The heatmap showed significantly up-regulated genes in cells expressing mutant SHP2, including *ASNS*, *PSAT1*, *PHGDH*, *SHMT2*, and *ALDH18A1* (**Figure 4B**). The FPKM values of 13 mutually dysregulated genes are shown in **Table 2**. Phosphoglycerate dehydrogenase (PHGDH) catalyzes the reversible oxidation of 3-phosphoglycerate to 3- phosphohydroxypyruvate, the first step of the *de novo* serine biosynthesis pathway. Subsequently, 3- phosphohydroxypyruvate is converted to phosphoserine by phosphoserine aminotransferase 1 (PSAT1) and then to serine by phosphoserine phosphatase. Serine hydroxymethyltransferase (SHMT2) catalyzes the reversible transition from serine to glycine and promotes the production of one-carbon units. Asparagine synthetase (ASNS) converts aspartate and glutamine to asparagine. *ALDH18A1* is a member of the aldehyde dehydrogenase family, and its encoded protein catalyzes the reduction of glutamate to delta1-pyrroline-5-carboxylate, a critical step in the *de novo* biosynthesis of proline, ornithine, and arginine. In addition, our analysis found carbon metabolism, as well as glycine, serine, and threonine metabolism pathways also dysregulated in SHP2-mutant cells, and the differentially expressed genes were also mainly *PSAT1*, *PHGDH*, and *SHMT2* (**Supplementary Figure 3**). These data suggest that the gain-of-function SHP2 mutants could promote serine and glycine synthesis *via* up-regulating the mRNA expression of *PSAT1*, *PHGDH*, and *SHMT2.*

**Table 2:** FPKM values of 13 genes commonly dysregulated in biosynthesis of amino acids pathway in HCD-57 cells expression SHP2-D61Y and -E76K.

Symbol	*ENO1^1^*	*ALDOA*	*PGAM1*	*ENO1^2^*	*CTH*	*PYCR1*	*SDSL*
HCD-57	75.26	122.95	18.87	55.07	0.80	1.86	7.60
SHP2-D61Y	633.94	1349.77	239.24	527.25	15.18	8.98	36.05
SHP2-E76K	195.85	281.77	56.60	136.91	5.30	4.64	20.00
Symbol	*GPT2*	*ALDH18A1*	*SHMT2*	*PHGDH*	*PSAT1*	*ASNS*	
HCD-57	2.72	36.96	65.34	61.91	48.00	104.82	
SHP2-D61Y	13.12	88.33	159.39	231.75	192.18	318.86	
SHP2-E76K	7.11	100.42	165.38	183.02	169.66	305.13	

ENO1^1^, Enolase 1, alpha non-neuron; ENO1^2^, Enolase 1B, retrotransposed.

## Discussion

In this study, we performed RNA-seq transcriptome sequencing analysis to identify dysregulated metabolic pathways and key genes based on HCD-57 cells transformed by SHP2-D61Y or -E76K. We found that DEGs caused by the expression of mutant SHP2 were mainly enriched in metabolic pathways, especially glutathione metabolism and biosynthesis of amino acids pathways. Importantly, we found that the biosynthesis of amino acids pathway was significantly activated in HCD-57 cells expressing SHP2-D61Y and SHP2-E76K. In addition, our data showed that the mRNA expression of *ASNS, PHGDH, PSAT1*, and *SHMT2* involved in asparagine, serine, and glycine biosynthesis were significantly increased in cells expressing mutant SHP2. Furthermore, our analysis found that *PSAT1*, *PHGDH*, and *SHMT2* were also key genes leading to the upregulation of carbon metabolism, as well as glycine, serine, and threonine metabolism pathways. These findings suggest that gain-of-function mutants of SHP2 might promote serine synthesis by activating the expression of *PSAT1* and *PHGDH*, and promote glycine biosynthesis by activating the expression of *SHMT2* for leukemia initiation and progression.

Reprogramming of metabolic pathways ensures the survival and proliferation of cancer cells in a nutrient-deficient environment (20). Besides, immune cell metabolic reprogramming alters immune cell function by interfering with critical transcriptional and post-transcriptional activation mechanisms, to keep growing tumors from being attacked by the immune system (20, 21). Alterations in carbohydrate metabolism in tumor cells have been reported. Tumor cells take up and use more glucose than they need, which is known as the Warburg effect (22). Recently, the amino acid dependence of tumor cells has received more and more attention (23). Amino acids have been demonstrated to be the dominant nitrogen source for hexosamines, nucleotides, and other nitrogenous compounds in rapidly proliferating cells (24, 25). Indeed, like glucose, there are major differences in the uptake and secretion of several amino acids in tumors relative to normal tissues. Compared to normal tissues, tumors require a large number of amino acids for bioenergetic, biosynthetic, and redox balance support (26, 27). This high demand is not limited to essential amino acids, but also for nonessential amino acids (NEAA) (24, 27). NEAA are not only components of proteins but also intermediate metabolites fueling multiple biosynthetic pathways. For example, glycine is synthesized from serine, threonine, choline, and hydroxyproline, and is degraded through the glycine cleavage system, serine hydroxymethyltransferase, and conversion to glyoxylate (28). In addition, glycine is utilized for the biosynthesis of glutathione, heme, creatine, nucleic acids, and uric acid (28).

The serine synthesis pathway (SSP) has been widely reported as a critical pathway enabling cancer cell proliferation and metastasis. Serine is a central precursor of biosynthetic metabolism, including being charged onto transfer RNAs for protein synthesis, providing head groups for sphingolipid and phospholipid synthesis, and serving as a precursor for cellular glycine and one-carbon unit (29). PHGDH is a rate-limiting enzyme for *de novo* serine biosynthesis and is mainly up-regulated to active serine biosynthesis. A high PHGDH expression has been extensively reported in several tumors, particularly breast and melanoma, and its high expression in these tumors is associated with poor prognosis (27). Importantly, its knockdown and silence exhibit obvious anti-tumor responses both *in vitro* and *in vivo* (30). PSAT1 is the transaminase for serine. It catalyzed the phosphohydroxypyruvate oxidized by PHGDH to produce phosphoserine, which is then dephosphorylated by 1-3-phosphoserine phosphatase (PSPH) to form serine. *PSAT1* expression is elevated in colon cancer and lung adenocarcinoma, and has been shown to enhance cell proliferation, metastasis, and chemoresistance (31, 32).

Serine and glycine metabolism are closely linked, as glycine is directly generated from serine *via* the serine hydroxymethyltransferase enzymes SHMT1 and SHMT2 (24). Importantly, the conversion of serine to glycine provides one-carbon units, which provide the necessary proteins, nucleic acids, lipids, and other biological macromolecules to support tumor growth (27). Serine, glycine, and their relation to one-carbon metabolism are highly relevant aspects of tumor metabolism (33). The directionality of serine/glycine conversion is a significant factor for cancer cell metabolism and evidence indicates that mitochondrial SHMT2 is the main serine-glycine converting enzyme (34). SHMT2 is upregulated in various cancer cells, and its depletion could trigger ROS-dependent mitochondria-mediated apoptosis (35).

ASNS converts aspartate and glutamine to asparagine and glutamate through an ATP-dependent amidotransferase reaction (36). Asparagine plays a crucial regulatory role in conditions of glutamine depletion (37). The precise role of asparagine in modulating tumor growth is unknown (38). ASNS is frequently up-regulated in tumors and is associated with poor prognosis (37, 39). In acute lymphoblastic leukemia (ALL), primary cells and many ALL cell lines exhibit a low expression level of ASNS (40). Su et al. found that different cells and patients expressed different amounts of ASNS mRNA and suggested it should pay attention to the differentiation of mRNA, protein content, and kinase activity in ASNS (41). Besides, Hutson et al. demonstrated that ASNS mRNA content increased in cells deprived of free amino acids (42). Later studies have also shown that endoplasmic reticulum stress increases ASNS transcription *via* the unfolded protein response (43). Ye et al. concluded that activation of ASNS by ATF4 with amino acid limitation may serve a vital biological process for tumor initiation and growth (44).

Exploring the metabolic adaptation mechanisms of uncontrolled tumor proliferation led by driver mutations has become a hot topic in cancer research. Preclinical research and clinical practice have shown therapeutic benefits by targeting tumor amino acid metabolism. One example is asparaginase that depletes both asparagine and glutamine in serum, which has been widely used to treat childhood acute lymphoblastic leukemia (45). A detailed understanding of the metabolic adaptation mechanisms of tumor cells may help the discovery of novel therapeutic targets, especially for relapsed and refractory neoplasms including SHP2-mutant JMML. The identification of specific driver mutation-dependent metabolic vulnerabilities is the bottleneck for the precise tumor treatment, which requires further investigation in the further.

## Data availability statement

The data presented in the study are deposited in the SRA repository, accession number PRJNA821339.

## Author contributions

YZ, ZP, CC, and YC conceived the project. YZ, ZC, CH, and YG performed the experiments. YZ, QZ, and DZ analyzed data. YZ, ZC, BH, ZP, CC, and YC wrote the manuscript. All authors contributed to the article and approved the submitted version.
