# Supplementary material for: Transcriptome analysis reveals effects of leukemogenic SHP2 mutations in biosynthesis of amino acids signaling
Source: Front Oncol. 2023 Jan 30;13:1090542. doi: 10.3389/fonc.2023.1090542 (PMC9922838; doi:10.3389/fonc.2023.1090542)

Supplementary Information

**
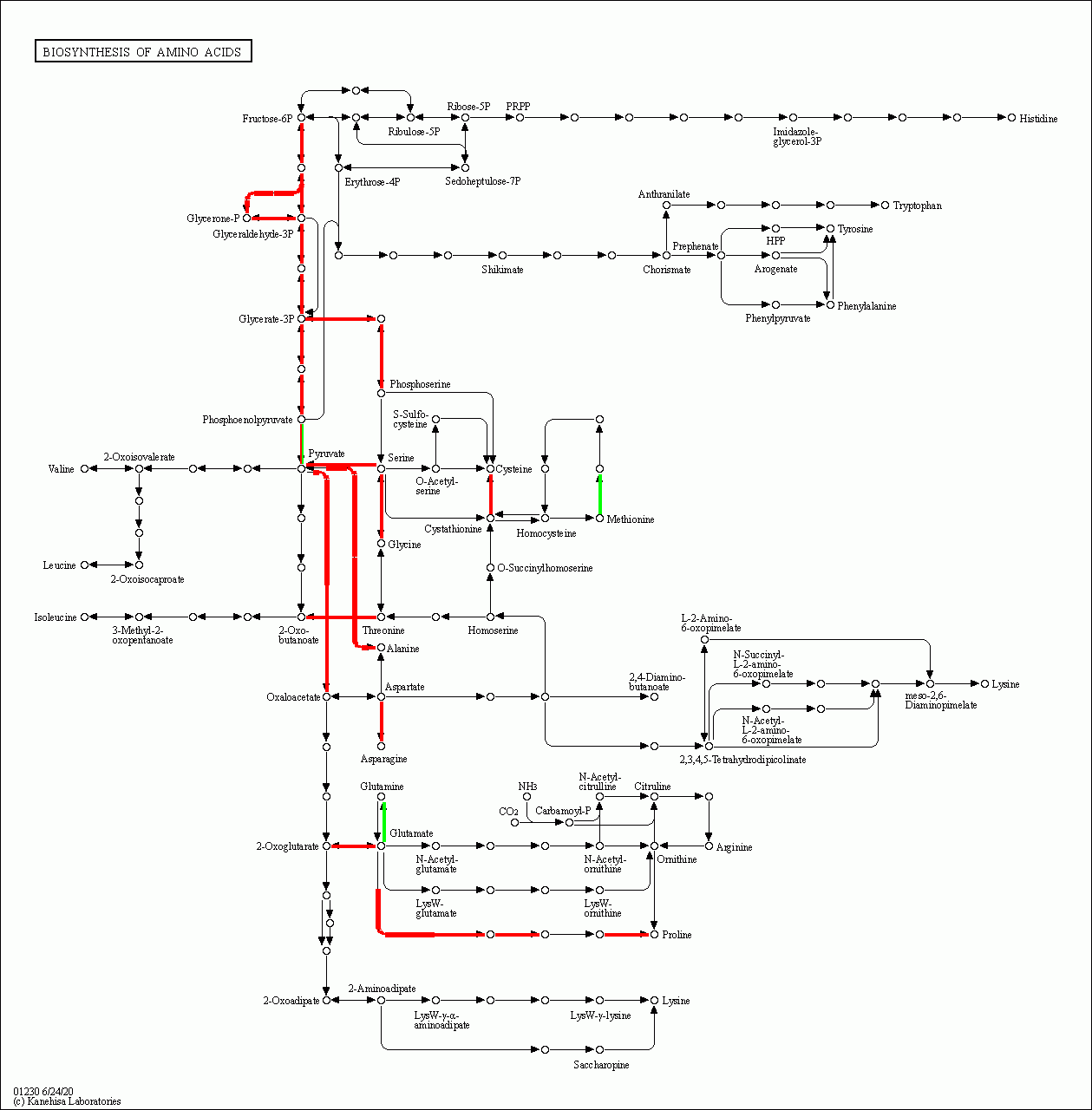
**

**Fig S1**. KEGG pathway analysis of biosynthesis of amino acids signaling based on DEGs of SHP2-D61Y compared with parental cells. The red lines represent up-regulated biological reactions and green represent down-regulated reactions.

**
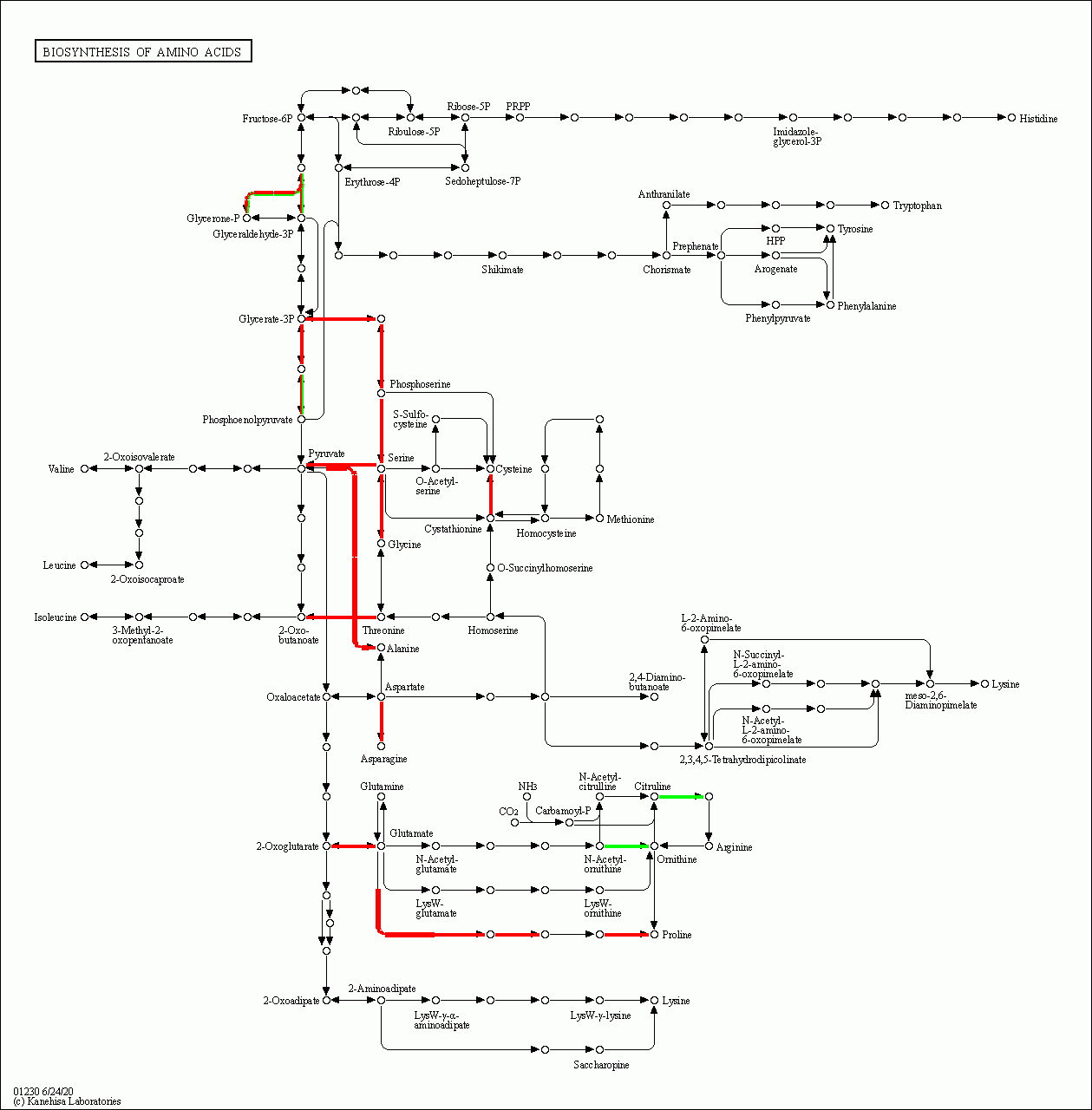
**

**Fig S2**. KEGG pathway analysis of biosynthesis of amino acids signaling based on DEGs of SHP2-E76K compared with parental cells. The red lines represent up-regulated biological reactions and green represent down-regulated reactions.

**Fig S3.** The expression of mutant SHP2 activated carbon metabolism and glycine, serine and threonine metabolism pathways via up-regulated *PSAT1*, *PHGDH*, and *SHMT2*. Gene Set Enrichment Analysis (GSEA) for (A) Carbon metabolism and Glycine, (B) Biosynthesis of amino acids. Venn diagram of DEGs and heat map of common significantly DEGs involved in (C) Carbon metabolism and (D) Serine and threonine metabolism pathway.


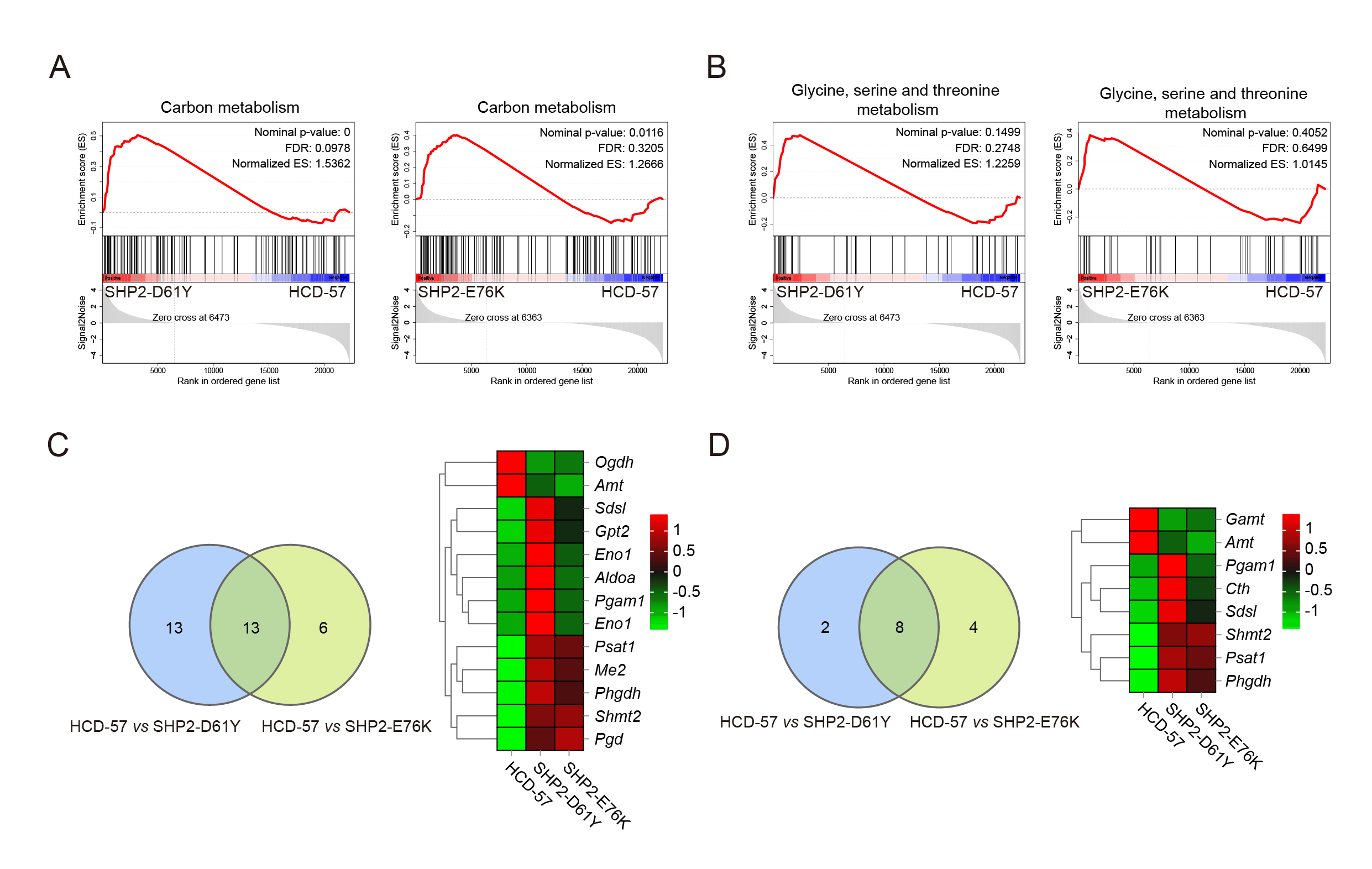

Supplement: Supplementary file 1 [file DataSheet_1.docx]
